# Network analysis of multimorbidity and health outcomes among persons with spinal cord injury in Canada

**DOI:** 10.3389/fneur.2023.1286143

**Published:** 2024-01-05

**Authors:** Nader Fallah, Heather A. Hong, Di Wang, Suzanne Humphreys, Jessica Parsons, Kristen Walden, John Street, Raphaele Charest-Morin, Christiana L. Cheng, Candice J. Cheung, Vanessa K. Noonan

**Affiliations:** ^1^Praxis Spinal Cord Institute, Vancouver, BC, Canada; ^2^Department of Medicine, University of British Columbia, Vancouver, BC, Canada; ^3^International Collaboration on Repair Discoveries, University of British Columbia, Vancouver, BC, Canada; ^4^Department of Orthopaedics, Vancouver Spine Surgery Institute, University of British Columbia, Vancouver, BC, Canada

**Keywords:** spinal cord injury, machine learning, network analysis, multimorbidity, outcomes

## Abstract

**Introduction:**

Multimorbidity, defined as the coexistence of two or more health conditions, is common in persons with spinal cord injury (SCI). Network analysis is a powerful tool to visualize and examine the relationship within complex systems. We utilized network analysis to explore the relationship between 30 secondary health conditions (SHCs) and health outcomes in persons with traumatic (TSCI) and non-traumatic SCI (NTSCI). The study objectives were to (1) apply network models to the 2011–2012 Canadian SCI Community Survey dataset to identify key variables linking the SHCs measured by the Multimorbidity Index-30 (MMI-30) to healthcare utilization (HCU), health status, and quality of life (QoL), (2) create a short form of the MMI-30 based on network analysis, and (3) compare the network-derived MMI to the MMI-30 in persons with TSCI and NTSCI.

**Methods:**

Three network models (Gaussian Graphical, Ising, and Mixed Graphical) were created and analyzed using standard network measures (e.g., network centrality). Data analyzed included demographic and injury variables (e.g., age, sex, region of residence, date, injury severity), multimorbidity (using MMI-30), HCU (using the 7-item HCU questionnaire and classified as “felt needed care was not received” [HCU-FNCNR]), health status (using the 12-item Short Form survey [SF-12] Physical and Mental Component Summary [PCS-12 and MCS-12] score), and QoL (using the 11-item Life Satisfaction questionnaire [LiSAT-11] first question and a single item QoL measure).

**Results:**

Network analysis of 1,549 participants (TSCI: 1137 and NTSCI: 412) revealed strong connections between the independent nodes (30 SHCs) and the dependent nodes (HCU-FNCNR, PCS-12, MCS-12, LiSAT-11, and the QoL score). Additionally, network models identified that cancer, deep vein thrombosis/pulmonary embolism, diabetes, high blood pressure, and liver disease were isolated. Logistic regression analysis indicated the network-derived MMI-25 correlated with all health outcome measures (*p* <0.001) and was comparable to the MMI-30.

**Discussion:**

The network-derived MMI-25 was comparable to the MMI-30 and was associated with inadequate HCU, lower health status, and poor QoL. The MMI-25 shows promise as a follow-up screening tool to identify persons living with SCI at risk of having poor health outcomes.

## Introduction

A spinal cord injury (SCI) occurs when the spinal cord is damaged either by trauma (e.g., car crash, falls; referred to as traumatic SCI [TSCI]) or through internal damage (e.g., degenerative, neoplastic, or infectious conditions; referred to as non-traumatic SCI [NTSCI]). The resulting injury can impair motor, sensory, and autonomic functions (1–4), and multiple body systems can be affected. Following a SCI individuals may experience complications such as spasticity, urinary tract infections, pneumonia, pressure injuries, and pain (5–7). More than 90% of persons living with SCI will experience at least one complication and more than half experience three or more complications which often require ongoing management (8).

The World Health Organization defines multimorbidity as the coexistence of two or more chronic health conditions in the same individual (9). Multimorbidity is a growing health concern as the population ages resulting in increased healthcare utilization (HCU) and poorer health outcomes (10). In 2014, Noonan et al. (11), developed a Multimorbidity Index (MMI) that assessed the presence of 30 secondary health conditions (SHCs), that included both complications following SCI and pre-existing comorbidities, and found that the MMI-30 significantly correlated with self-reported HCU using the 7-item HCU questionnaire (12), physical and mental health status as measured by the 12-item Short Form-12 (SF-12) Physical (PCS-12) and Mental Component Summary (MCS-12) scores, and quality of life (QoL) measured using Life Satisfaction-11 (LiSAT-11) first question and a single item QoL measure. A higher MMI score was associated with lower PCS-12 and MCS-12 scores, as well as significantly lower LiSAT-11 and overall QoL scores. More recently, the same MMI-30 was validated in persons with NTSCI and demonstrated similar relationships with HCU, PCS-12, MCS-12, LiSAT-11, and QoL scores (13).

Network analysis is a powerful tool used to visualize complex relationships among variables (i.e., nodes) and examine the importance of each variable in the network structure via connections (i.e., edges) (14). In healthcare, network analysis has been broadly applied to describe, explore, and understand structural and relational aspects of health. Examples include modelling disease outbreaks (15), resource utilization (16), as well as understanding multimorbidity (17). In SCI, the use of network analysis can identify important nodes and relationships among SHCs and health outcomes (18).

Depending on the data type, certain statistical models can be applied to create the network model. The pairwise Markov Random Field (MRF) is a broad class of statistical modelling, characterized by undirected edges between nodes that indicate conditional dependence between nodes (19). Common examples of MRF include the Gaussian Graphical Model (GGM) for continuous normally distributed data (20) and ordinal data (21); the Ising Model for binary data (22); and the Mixed Graphical Model (MGM) for mixed data consisting of both categorical and continuous variables (23). Within these networks, an undirected edge reflects an association between two nodes, and the edge weighted reflects a quantitative value which indicates the reliability of the interaction.

Network centrality provides insight into the relative importance of each node in the context of the other nodes in the network by assigning a score to each node. Different centrality indices, such as strength, closeness, or betweenness, can provide insights into different dimensions of centrality (14). High centrality nodes have strong connections to many other nodes, and act as hubs that connect otherwise disparate nodes to one another. Low centrality nodes exist on the periphery of the network, and have fewer and weaker connections to other nodes within the network (14). Thus, the network properties can help identify relevant sub-structures within a network and inform which nodes to target, thereby creating a more concise screening tool for determining connections between medical diagnoses and health outcomes.

In this study, network analysis was used to explore the relationships between the 30 SHCs included in the MMI-30 with HCU, health status (PCS-12, MCS-12), and QoL (LiSAT-11, QoL score) in persons with SCI, with the intent to refine the MMI-30 for clinical use. Specifically, the objectives were to (1) apply three network models (GGM, Ising, and MGM) to the 2011–2012 Canadian SCI Community Survey dataset (24, 25) to identify key variables important in each network, (2) create a short form of the MMI-30 using network analysis, and (3) compare the network-derived MMI to the MMI-30 in persons with TSCI and NTSCI.

## Materials and methods

### Data source

This study used the 2011–2012 Canadian SCI Community Survey data, described in full by Noreau et al. (24, 25). In brief, the survey was designed to better understand the service-related needs, service utilization and health outcomes in persons with TSCI and NTSCI living in the community.

### Measures

The 2011–2012 Canadian SCI Community Survey data included self-reported personal (e.g., age, sex), injury (e.g., level, completeness, and type of SCI), and environmental factors (e.g., living setting). The level and completeness of SCI was determined indirectly using the participants’ answers about their lesion and sensorimotor and mobility capabilities and classified according to the American Spinal Cord Injury Association (ASIA) Impairment Scale (AIS) as per the International Standards for Neurological Classification of SCI (ISNCSCI) (25).

#### Multimorbidity

Participants were asked about the presence or absence of 30 SHCs within the past 12 months (Supplementary Table 1). The SHCs included comorbidities (present prior to the SCI) and secondary complications following the injury. Participants who answered, “do not know” were considered as “do not have the condition.” The MMI was the sum of 30 SHCs, ranging from 0 to 30, a higher score indicating more SHCs present (11).

#### Healthcare utilization

Participants reported their HCU within the past 12 months using the 7-item Health Care Utilization questionnaire (12). HCU included contact with healthcare professionals (HCP), the number of HCP seen, the number of visits, type of HCP, rehospitalization, and hospital length of stay. Furthermore, participants were asked “During the past 12 months, was there ever a time when you felt that you needed healthcare but did not receive it?.” If “Yes,” participants were classified as “felt needed care was not received” (FNCNR), and asked to report the frequency, type of care was needed but not received, and the reason for not receiving care. If “No,” participants were classified as “felt needed care was received” (FNCR) (13). In this study the response to the HCU related to FNCNR (HCU-FNCNR) was used in the analysis.

#### Health status

The SF-12 was included to measure physical and mental health status (26, 27). The SF-12 measures eight health domains to provides a PCS-12 and a MCS-12 score (26). For PCS-12, a score of ≤50 has been recommended as a cut-off to determine a physical condition, while a score of ≤42 on the MCS-12 may be indicative of mental health conditions (28).

#### Quality of life

Regarding the assessment of QoL, two distinct measures were employed. First, the LiSAT-11 measures satisfaction in 10 specific domains as well as overall life satisfaction asking about “My life as a whole is.” Respondents were asked to rate their satisfaction levels on a scale ranging from “very dissatisfied” (coded as 1) to “very satisfied” (coded as 6) (11). In this study just the first question asking about overall life satisfaction was used. Second, the 5-point single-item QoL measure, “How do you rate your overall QoL?,” where 1 is rated as “poor” is rated as 5 being “good,” was used (11). To simplify the analysis, the LiSAT-11 responses were dichotomized. Responses falling within the range of 1–4 were categorized as “not satisfied” (coded as 0), while those rated 5–6 were classified as “satisfied” (coded as 1) (11). Similarly, the single-item QoL measure on a scale of 1 (poor) to 5 (good) was coded as “not satisfied”/“poor” (coded as 0) or “satisfied”/“good” (coded as 1) (11).

### Network analysis

Three weighted undirected biological networks were constructed using the GGM (for continuous data with multivariate gaussian distribution), Ising Model (for binary variables), and MGM (for mixed data with continuous and discrete variables). Depending on the type of network, nodes represented the 30 SHCs and the health outcome measures (HCU-FNCNR, PCS-12, MCS-12, LiSAT-11, and QoL scores), and edges represented the relationships between these nodes. The edge weight or partial correlation coefficients, which ranged from −1 to 1, represented the conditional independence associations. To enhance prediction accuracy and interpretability of the models, the L1 logistic Least Absolute Shrinkage and Selection Operator (LASSO) regression was applied to each node to estimate the connections between the node and other nodes (i.e., neighbor sets) (29). The Extended Bayes Information Criterion (EBIC) was also used to choose the best neighbor set with the lowest EBIC (29). Furthermore, the hyperparameter γ determined model sparsity, a higher γ led to a smaller number of false positives and therefore a sparser network.

In addition, three centrality measures were performed (i.e., descriptive statistics of a nodes’ influence and its role in the network). Strength centrality is the absolute sum of a nodes’ edge weights, a higher value or *Z* score indicates a stronger connection. Expected Influence (EI) is a node’s importance in activating or deactivating other nodes in the network that have negative edges, greater *Z* scores indicate influential nodes (30). Betweenness centrality is the number of times a node is in the shortest path between two other nodes which represents its role in connecting the communities of nodes.

The GGM shows which variables predict one-another, allowing for sparse modeling of covariance structures, and may highlight potential causal relationships between observed variables (31). It estimates a network of partial correlation coefficients (i.e., the correlation between two variables after conditioning on all other variables in the dataset) (32). In the Ising Model, continuous variables such as PCS-12, MCS-12, and age were removed when fitting this model. In contrast, in the MGM, direct associations between heterogenous variables and the joint probability density allowed arbitrary probabilistic questions of the data to be explored (33).

For additional information comparing the three network models and their reliability, please see Supplementary Material Section 2.

### Statistical analyses

To compare TSCI and NTSCI, descriptive and bivariate analyses were performed using the Chi-square test (Fisher’s exact test if the expected cell counts were less than five) or T-test (Mann–Whitney U-test for non-normal data), and depending on the data distribution, either the Pearson or Spearman correlation were used. Both statistically significant and clinically relevant factors (e.g., age, sex, incomplete SCI, and the MMI) were included in regression models to examine their effect on the measures (HCU-FNCNR, PCS-12, MCS-12, LiSAT-11, and the QoL score). For PCS-12 and MCS-12 (continuous variables) multiple linear regression models were used, and for HCU-FNCNR, LiSAT-11, and the QoL score (categorical variables) logistic regression models were used. Further side-by-side comparisons of the network-derived MMI and the MMI-30 were performed. All statistical analyses were performed using SAS software, Version 9.4 of the SAS System for Windows (Copyright © 2013, SAS Institute Inc., Cary, NC.). Value of *p*s <0.05 were considered statistically significant. Networks were estimated and visualized using RStudio (version 3.4) using the “bootnet” package (CRAN; https://cran.r-project.org/) (32).

## Results

### Baseline participant characteristics

Of 1,549 participants, 1,137 (73.4%) were participants with TSCI and 412 (26.6%) were participants with NTSCI. Table 1 summarizes the demographic, clinical, and outcome comparisons between participants with TSCI and NTSCI, as described in the paper by Noreau et al. (24). Age at injury, sex, AIS, and lesion severity significantly differed among participants with TSCI and NTSCI. In response to HCU question “During the past 12 months, was there ever a time when you felt that you needed healthcare but did not receive it?” (i.e., HCU-FNCNR), in total, 292 (25.7%) and 89 (21.7%) participants with TSCI and NTSCI, respectively, answered “yes” to feeling needed care was not received (Table 1).

**Table 1:** Demographic and clinical characteristics of participants with traumatic spinal cord injury (TSCI) and non-traumatic spinal cord injury (NTSCI), adapted from Noreau et al. (24, 25), Noonan et al. (11), and Hong et al. (13).

Variables	TSCI *n* = 1,137	NTSCI *n* = 412	*p* value
Age at injury, years (mean, SD)*	48.3 ± 13.3	53.1 ± 14.9	**<0.001**
Years since injury, (mean, SD)*	18.5 ± 13.1	18.7 ± 17.1	NS
Sex, male (*n*, %)*	806 (70.9)	235 (57.0)	**<0.001**
Ethnicity (Caucasian), *n* (%)*	1,052 (92.5)	377 (91.5)	NS
Region of residence (*n*, %)*			
Quebec	275 (24.2)	121 (29.4)	NS
Ontario	245 (21.5)	101 (24.5)	
British Columbia	227 (20.0)	69 (16.7)	
Other (Prairies and Atlantic provinces)	390 (34.3)	121 (29.4)	
Self-reported current neurological classification (*n*, %)*^#^			
Tetraplegia AIS A or B	229 (21.3)	14 (3.7)	**<0.001**
Paraplegia AIS A or B	361 (33.6)	81 (21.4)	
Tetraplegia AIS C or D	301 (28)	69 (18.2)	
Paraplegia AIS C or D	184 (17.1)	215 (56.7)	
Missing	62	33	
Lesion severity (*n*, %)*			
Complete	444 (39.1)	72 (17.5)	**<0.001**
Incomplete	693 (61)	340 (82.5)	
Area of residence (population)			
<10,000	244 (21.9)	77 (19.5)	NS
10,000–100,000	196 (17.6)	53 (13.4)	
>100,000	431 (38.7)	176 (44.6)	
Large cities	242 (21.7)	89 (22.5)	
Missing	24	17	
Education level*			
Less than high school	157 (13.8)	59 (14.3)	NS
High school	249 (22)	87 (21.3)	
College/university	561 (49.6)	205 (50.1)	
Graduate studies	92 (8.1)	27 (6.6)	
Others	73 (6.5)	31 (7.6)	
No record	5	3	
Marital status*			
Married	466 (41.2)	181 (44.9)	NS
Common-law	107 (9.5)	43 (10.7)	
Widowed, separated or divorced	205 (18.1)	77 (19.1)	
Single, never married	353 (31.2)	102 (25.3)	
Undeclared	6	9	
Current living setting*			
Own home	793 (70)	256 (63.8)	NS
Rental housing	233 (20.6)	99 (24.7)	
Assisted-living	24 (2.1)	13 (3.2)	
Hospital/long-term care facility	17 (1.5)	5 (1.2)	
Others	66 (5.8)	28 (7)	
Missing	4	11	
Multimorbidity measure, mean (SD)^†^			
MMI-30	13.1 (4.3)	12.4 (4.9)	**0.015**
Health outcome measures, mean (SD)^†^			
PCS-12 score	33.5 (8.6)	33.5 (8.5)	NS
MCS-12 score	51.6 (11.4)	48.5 (11.6)	**<0.001**
Life Satisfaction-11, question 1 score	4 (1)	3.9 (1)	NS
Overall QoL score	3.8 (0.9)	3.7 (0.9)	**0.001**
Had a contact with an HCP, yes, *n* (%)	1,017 (89.4)	360 (87.3)	NS
Number of HCPs seen, mean (SD)	3.9 ± 2.5	4 ± 2.5	NS
Frequency of any HCP seen, mean (SD)	44.4 ± 138	47.5 (101.7)	NS
Re-hospitalized, yes, *n* (%)	297 (26.1)	103 (25)	NS
Number of nights spent in hospital, mean (SD)	23.5 (46.7)	27.4 (47.3)	NS
Felt needed care was not received, yes, *n* (%)	292 (25.7)	89 (21.7)	NS
Number of times needed care could not be received, mean (SD)	9.8 (35.7)	24.2 (112.6)	**0.023**

*Demographic and clinical characteristics between persons with TSCI or NTSCI adapted from Noreau et al. (24, 25).^†^Multimorbidity, health status, and healthcare utilization in the past 12 months for persons with TSCI and NTSCI adapted from Noonan et al. (11) and Hong et al. (13), respectively.^#^The American Spinal Injury Association (ASIA) Impairment Scale (AIS) was evaluated indirectly from participants’ answers about their lesion and sensorimotor and mobility capabilities. Bold font indicates statistical significance.AIS, American Spinal Injury Association (ASIA) Impairment Scale; MMI-30, Multimorbidity Index consisting of 30 secondary health conditions; SF-12, Short Form 12-item survey; PCS-12, Physical Component Summary score; MCS-12, Mental Component Summary score; HCP, healthcare professional, LiSAT-11, Life Satisfaction-11; QoL, Quality of Life measure; SD, standard deviation; NS, not significant.

### Bivariate analysis

The Supplementary Table 1 shows hypothesis testing of the 30 SHCs for the health outcome measures: HCU-FNCNR, PCS-12, MCS-12, LiSAT-11 and the QoL scores in persons with TSCI and NTSCI. In the TSCI dataset, only deep vein thrombosis/pulmonary embolism (DVT) and diabetes did not significantly differ across any of the measures. For the NTSCI dataset, cancer, DVT, diabetes, high blood pressure and liver disease did not significantly differ across any of the measures. All other SHCs had significant associations with the health outcome measures (*p* < 0.05).

### Network analysis

#### Gaussian graphical model

**Figure 1:**
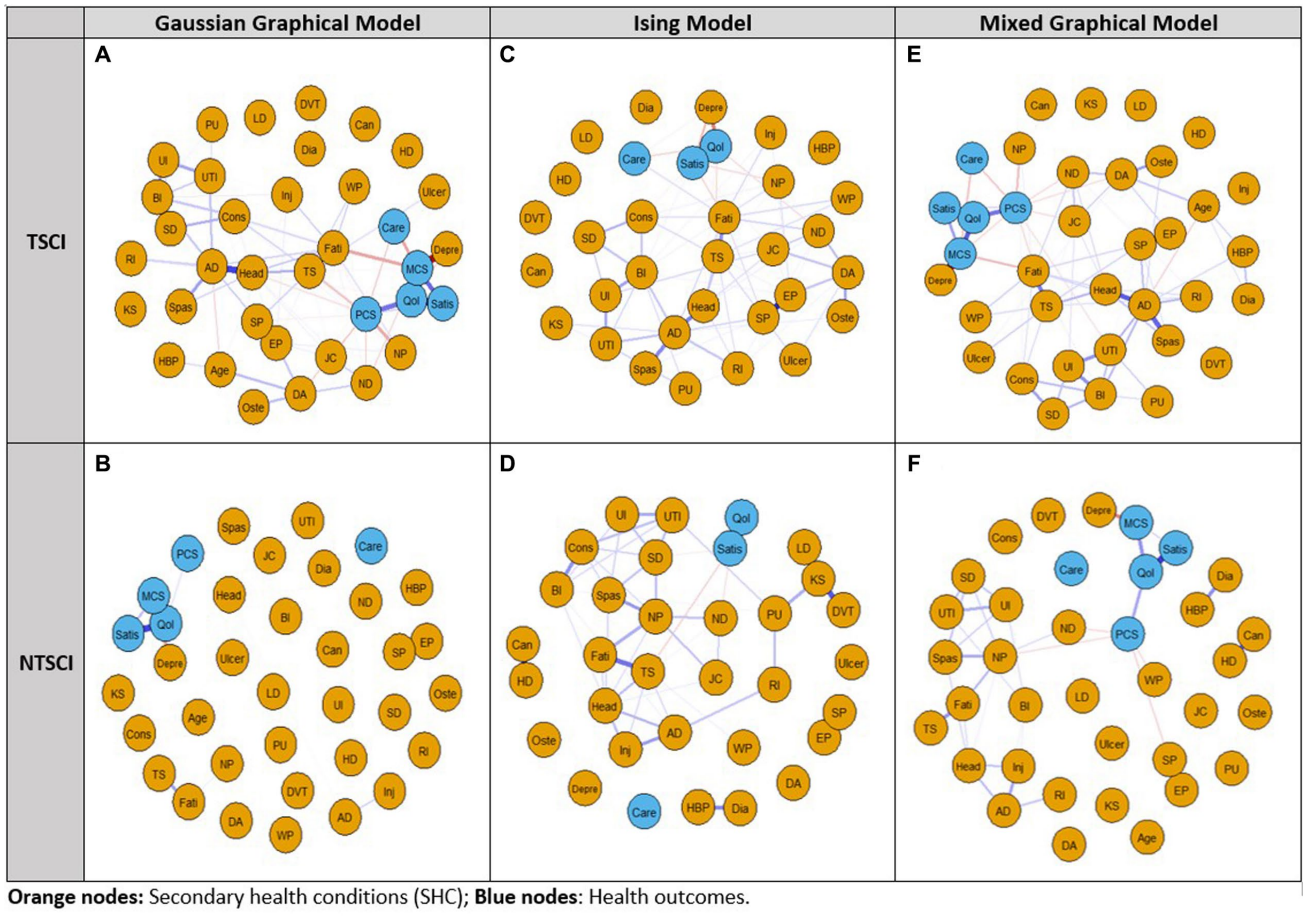
Network analysis of the 2011–2012 Canadian SCI Community Survey dataset using the Gaussian Graphical Model, Ising Model, and Mixed Graphical Model in persons with traumatic spinal cord injury (TSCI: **A,C,E**) and non-traumatic spinal cord injury (NTSCI: **B,D,F**). Nodes represent the 30 secondary health conditions (SHCs, orange dots) and health outcome measures (blue dots). Edges (lines) represent a temporal/contemporaneous relationship between another variable at the next measurement. Blue edges have positive associations and red edges have negative associations; edge intensity represents the strength of the relationship; stronger associations are more saturated. For the Ising Model independent non-binary variables PCS-12, MCS-12, and age were removed. SHCs consisted of AD, Autonomic dysreflexia; BI, Bowel incontinence; Can, Cancer; Cons, Constipation; DA, Osteoarthritis/degenerative arthritis; Depre, Depression/mood problem; Dia, Diabetes; DVT, Deep vein thrombosis/pulmonary embolism; EP, Elbow/wrist problems; Fati, Fatigue; HBP, High blood pressure; HD, Heart disease; Head, Light headedness/dizziness; Inj, Injuries caused by loss of sensation; JC, Joint contractures; KS, Kidney stones; LD, Liver disease; ND, Neurological deterioration; NP, Neuropathic pain; Oste, Osteoporosis; PU, Pressure ulcers; RI, Respiratory infections; SD, Sexual dysfunction; SP, Shoulder problems; Spas, Spasticity; TS, Trouble sleeping; Ulcer, Ulcer/gastric esophageal reflux disease; UI, Urinary incontinence; UTI, Urinary tract infection; WP, Weight problem. Health outcome measures included Care: felt needed care not received (Y/N); PCS-12, Physical Component Summary score; MCS-12, Mental Component Summary score; QoL, Quality of Life; Satis, Life Satisfaction-11.

**Figure 2:**
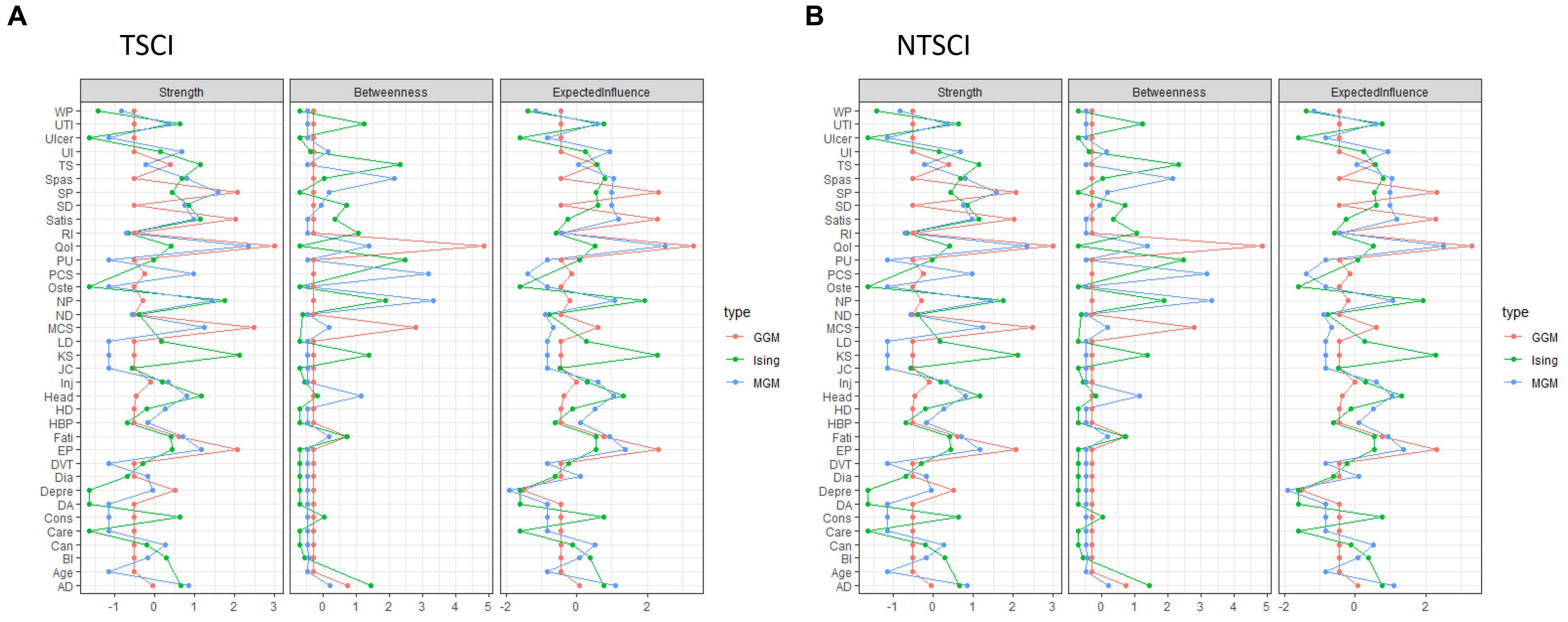
The centrality indices: strength, betweenness, and expected influence for each network model: Gaussian Graphical Model (GGM), Ising Model (Ising), and Mixed Graphical Model (MGM) in person with **(A)** traumatic spinal cord injury (TSCI) and **(B)** non-traumatic spinal cord injury (NTSCI). Nodes represent the 30 secondary health conditions (SHCs, i.e., multimorbidity) and health outcome measures. SHCs consisted of AD, Autonomic dysreflexia; BI, Bowel incontinence; Can, Cancer; Cons, Constipation; DA, Osteoarthritis/degenerative arthritis; Depre, Depression/mood problem; Dia, Diabetes; DVT, Deep vein thrombosis/pulmonary embolism; EP, Elbow/wrist problems; Fati, Fatigue; HBP, High blood pressure; HD, Heart disease; Head, Light headedness/dizziness; Inj, Injuries caused by loss of sensation; JC, Joint contractures; KS, Kidney stones; LD, Liver disease; ND, Neurological deterioration; NP, Neuropathic pain; Oste, Osteoporosis; PU, Pressure ulcers; RI, Respiratory infections; SD, Sexual dysfunction; SP, Shoulder problems; Spas, Spasticity; TS, Trouble sleeping; Ulcer, Ulcer/gastric esophageal reflux disease; UI, Urinary incontinence; UTI, Urinary tract infection; WP, Weight problem. Health outcome measures included Care: healthcare utilization-felt needed care not received (coded as Y/N); health status: PCS Physical Component Summary (PCS-12) score and MCS Mental Component Summary (MCS-12) score; QoL, Quality of Life; Satis, Life Satisfaction-11.

In the TSCI dataset, the GGM showed that five nodes (liver disease, DVT, cancer, heart disease and kidney stones) were independent, i.e., missing an edge (Figure 1A). The strongest connections were between depression and MCS-12 (edge weight 0.3), elbow/wrist problems and shoulder problems (edge weight 0.297), and the QoL score and LiSAT-11 (edge weight 0.282). The network structure also indicated that (1) HCU-FNCNR (labeled as “Care”) negatively correlated with PCS-12, MCS-12 and the QoL score, and positively correlated with light headedness/dizziness and fatigue; (2) the QoL score negatively correlated with depression, light headedness/dizziness, HCU-FNCNR and trouble sleeping, and positively correlated with LiSAT-11, PCS-12 and MCS-12; and (3) LiSAT-11 positively correlated with the QoL score, MCS-12 score and PCS-12 score, and negatively correlated with neuropathic pain. Overall, the GGM had a medium stability of estimation, strength and edge weight had centrality stability (CS)-coefficient values >0.5, and estimations of EI were unstable. The centrality indices: strength, betweenness, and EI for the TSCI GGM indicated that MCS-12 had the strongest strength, autonomic dysreflexia (AD) had the highest betweenness, and both AD and the QoL score had high EI (Figure 2A). HCU-FNCNR had lower strength and EI was significantly different from around one third of the nodes. The QoL score ranked first in betweenness, while most nodes had zero betweenness, including the other four health outcome measures (HCU-FNCNR, PCS-12, MCS-12, and LiSAT-11 scores).

For the NTSCI dataset, the GGM network structure was sparse (Figure 1B). Only one third of the nodes were connected and the connections were weak. The strongest connections were between elbow/wrist problems and shoulder pain (edge weight 0.272), followed by the QoL score and LiSAT-11 (edge weight 0.199), and the QoL score and MCS-12 (edge weight 0.14). Additionally, depression and MCS-12 had an edge weight of−0.107, which was significantly different from all other edges. Notably, HCU-FNCNR was not connected to any other nodes, and had zero strength, betweenness and EI. LiSAT-11 was positively associated with the QoL score and MCS-12, and the QoL score positively correlated with PCS-12, MCS-12, and LiSAT-11. All centrality measures and edge weights indicated an unstable estimation, the CS-coefficients were < 0.5. The QoL score and LiSAT-11 had a significantly larger strength and EI than around half of the nodes, the QoL score also had the largest betweenness. MCS-12 had a significantly higher strength than around two thirds of the nodes and a medium EI that was only significantly different from that of LiSAT-11, the QoL score, shoulder pain, and elbow/wrist pain. Moreover, the PCS-12 had a low strength and EI that was significantly different from less than 10 nodes.

#### Ising model

In the TSCI dataset, the Ising Model showed six independent nodes (cancer, liver disease, DVT, high blood pressure, heart disease, and diabetes) (Figure 1C). The strongest connections were between the QoL score and LiSAT-11, elbow/wrist problems and shoulder problems, and the QoL score and depression with edge weights of 2.165, 1.795, and-1.08, respectively. Both the QoL score, and LiSAT-11 had a negative relationship with depression. A negative association was identified between the QoL score and HCU-FNCNR, trouble sleeping, neurological deterioration, and light headedness/dizziness. LiSAT-11 was negatively associated with depression, fatigue, neuropathic pain, constipation, and joint contractures. Additionally, HCU-FNCNR was negatively associated with the QoL score, but positively associated with depression and fatigue. Based on the CS-coefficient of strength, EI, and edge weight (all >0.5), AD had the strongest strength, betweenness and EI, suggesting that it had importance in the network and the strongest connection to other nodes. The QoL score and LiSAT-11 had high strength and medium EI, but the QoL score showed more significant differences than LiSAT-11 for both measures. HCU-FNCNR had a medium absolute value of strength and EI and the values were significantly different from around one third of the nodes.

In the NTSCI dataset, the Ising Model showed four nodes (degenerative arthritis/osteoarthritis, ulcer/gastric esophageal reflux disease, HCU-FNCNR, and osteoporosis) were independent (Figure 1D). Moreover, three node pairs were separate from the main network cluster, i.e., shoulder pain and elbow/wrist pain (edge weight 1.834), cancer and heart disease (edge weight 1.272), as well as high blood pressure and diabetes (edge weight 0.842). Within the main cluster, the strongest connection was between the QoL score and LiSAT-11 (edge weight 1.81), of which LiSAT-11 was also negatively associated with trouble sleeping, neurological deterioration, and sexual dysfunction. However, the network was unstable (all CS-coefficients were < 0.5). While kidney stones had the largest strength and EI, the significance test suggested that the strength and EI were not significantly different than that of other nodes. LiSAT-11 had a relatively large strength and a medium EI where the strength was only significantly different from four nodes and EI showed no significant differences from other nodes. The QoL score had a low strength and EI, and the significance test showed that the values were only different from very few nodes, four for strength and two for EI.

#### Mixed graphical model

For TSCI, the MGM showed cancer, kidney stone, liver disease, heart disease, DVT and injuries caused by loss of sensation (e.g., burns from carrying hot liquids in the lap or sitting too close to a heater or fire) were independent (Figure 1E). The strongest connection was between elbow/wrist problems and shoulder problems (edge weight 0.825). A negative correlation was apparent between HCU-FNCNR, PCS-12, and MCS-12, whereas LiSAT-11 was positively correlated with the QoL score, PCS-12, and MCS-12, and the QoL score was positively correlated with LiSAT-11, PCS-12, and PCS-12. The stability of edge weight, strength and EI were good (CS-coefficient > 0.5), while betweenness indicated instability (CS-coefficient 0.206) which may be caused by weak connections between nodes. Interestingly, AD had the strongest strength, betweenness and EI and was the most powerful node in the network (Figure 2A). Its strength and EI were significantly different from most nodes in the network. The node HCU-FNCNR (labeled as “Care”) had poor performance in all centrality indices, its strength and EI were significantly smaller than the other nodes. PCS-12 and MCS-12 had significantly larger strength, but medium EI, while the QoL score, and LiSAT-11 had significantly larger strength and EI.

For NTSCI, the MGM network was sparse (Figure 1F). Of the health outcome measures, HCU-FNCNR was independent. The strongest connections were between elbow/wrist problems and shoulder pain (edge weight 0.998), LiSAT-11 and the QoL score (edge weight 0.758), followed by cancer and heart disease (edge weight 0.61). LiSAT-11 was positively associated with MCS-12, and the QoL score was also positively associated with MCS-12 and PCS-12. Despite this, the three centrality measures and edge weight showed an unstable network (CS-coefficient < 0.5). The strength and betweenness of many nodes were estimated to be zero. The QoL score had the largest strength and EI, and neuropathic pain had the best performance for betweenness (Figure 2B). Depression had a medium EI but was significantly different from all other nodes except PCS-12 and MCS-12. LiSAT-11 had a medium strength and EI that were significantly different from a third of the nodes, while MCS-12 had significantly larger strength but lower EI. The PCS-12 also had significantly lower EI and larger strength; however, its strength was only significantly different from age and the QoL score.

### Comparison of network models between TSCI and NTSCI

The three network models between persons with TSCI and NTSCI presented a similar pattern (Figures 1, 2); however, the consistency of results among the three network analyses in the TSCI group was stronger. In terms of the edge weight, the Ising Model provided the largest overall edge weight, followed by the MGM and then the GGM.

For TSCI data, the three network structures showed similarities in terms of which nodes were connected and/or isolated. In general, the edge weights of the QoL score and LiSAT-11, MCS-12 and depression, and elbow/wrist problems and shoulder problems were strong in all models. Cancer, DVT, liver disease, and heart disease were isolated in all models. The estimation of strength was stable for all models. For AD, the magnitude of strength was slightly different among the methods, where MGM gave the highest value and GGM gave the lowest value. For EI, the estimation was stable under the Ising Model and MGM, but the CS-coefficient was 0.361 under the GGM. The estimation of EI for the QoL score was quite different between the Ising Model and the others. Betweenness indicated instability in all models, and the Ising Model had the lowest CS-coefficient. Moreover, edge weight was stable under all models.

For NTSCI data, all three network models were sparse. Only one-third of the nodes were connected, and the associations were generally weak. In all models, the connection between elbow/wrist and shoulder problems had the strongest edge. The QoL score and LiSAT-11, cancer and heart disease, MCS-12 and depression were also closely related. The Ising Model and MGM had zero CS-coefficients for all centrality indices (Figure 2B). Furthermore, the GGM presented an unstable strength, EI [CS (cor = 0.7) = 0.438], and betweenness [CS (cor = 0.7) = 0]. Meanwhile, the edge weight estimation was unstable under all three models. The estimation of strength was quite different among models. The differences among the models were larger than that for TSCI data, and GGM provided a larger magnitude compared with the others. For strength, the estimation of kidney stones was quite different among the models where the Ising Model gave the largest estimation. For EI, the estimated value of the QoL score was very different between the Ising Model and the other two where the Ising Model gave a much smaller number. The differences also existed for kidney stones where the Ising Model had a much bigger value. The estimation of betweenness was similar, however GGM gave a larger estimation for the QoL and MCS-12 scores, while Ising had a larger estimation for urinary tract infections and trouble sleeping.

Thus, by comparing the three network results and combining the bivariate analysis, cancer, DVT, diabetes, high blood pressure and liver disease were removed from the MMI-30, and the remaining 25 SHCs formed the network-derived MMI-25.

### Comparison of MMI-30 vs. MMI-25 using the TSCI dataset

To test the efficiency of the network-derived MMI-25 using the TSCI dataset, the MMI-30 and MMI-25 logistic regression model outcomes were compared for HCU-FNCNR, PCS-12, MCS-12, LiSAT-11, and the QoL score (Table 2). Both the MMI-30 and the MMI-25 significantly correlated with each of the health outcome measures (*p* < 0.0001), suggesting that the MMI-25 was as effective as the MMI-30.

**Table 2:** Comparison of MMI-30 vs. MMI-25 regression models and the health outcome measures in persons with traumatic spinal cord injury (TSCI).

A) Analysis of “Healthcare Utilization-Felt that Needed Care was Not Received” using logistic regression.								
Variables	MMI-30				MMI-25			
	β	value of p	OR	95% CI	β	value of p	OR	95% CI
Live in own home	-0.107	0.169	0.81	0.60, 1.10	−0.111	0.154	0.80	0.59, 1.09
Incomplete SCI	−0.006	0.931	0.99	0.74, 1.32	−0.007	0.924	0.99	0.74, 1.32
Sex, male	−0.257	**0.001**	0.60	0.44, 0.81	−0.256	**0.001**	0.60	0.45, 0.81
Age	−0.002	0.775	1	0.99, 1.01	0.000	1.000	1	0.99, 1.01
Days since injury	0.000	**0.021**	1	1, 1	0.000	**0.024**	1	1, 1
MMI	0.174	**<0.0001**	1.19	1.15, 1.24	0.182	**<0.0001**	1.20	1.15, 1.25

B) Analysis of “PCS-12” using multiple linear regression.						
Variables	MMI-30			MMI-25		
	*β*	Value of *p*	95% CI	*β*	Value of *p*	95% CI
Age	−0.080	**<0.0001**	−0.12, −0.05	−0.090	**<0.0001**	−0.13, −0.05
Sex, male	−0.989	0.057	−2.01, 0.03	−0.996	0.055	−2.01, 0.02
Incomplete SCI	0.273	0.571	−0.67, 1.22	0.284	0.556	−0.66, 1.23
Area of residence*						
Large cities	1.830	**0.010**	0.44, 3.22	1.854	**0.009**	0.47, 3.24
Pop >100 k	1.961	**0.002**	0.74, 3.19	1.977	**0.002**	0.75, 3.20
Pop 10 k-100 k	−0.460	0.540	−1.93, 1.01	−0.420	0.575	−1.89, 1.05
Not married	−1.078	**0.024**	−2.01, −0.14	−1.117	**0.019**	−2.05, −0.18
MMI	−0.858	**<0.0001**	−0.97, −0.75	−0.892	**<0.0001**	−1.00, −0.78

C) Analysis of “MCS-12” using multiple linear regression.						
Variables	MMI-30			MMI-25		
	*β*	Value of *p*	95% CI	*β*	Value of *p*	95% CI
Age	0.039	0.119	−0.01, 0.09	0.031	0.224	−0.02, 0.08
Sex, male	0.874	0.225	−0.54, 2.29	0.887	0.220	−0.53, 2.30
Incomplete SCI	−1.067	0.111	−2.38, 0.25	−1.036	0.123	−2.35, 0.28
Area of residence*						
Large cities	−0.012	0.990	−1.94, 1.92	−0.001	0.999	−1.93, 1.93
Pop >100 k	0.423	0.625	−1.28, 2.12	0.426	0.624	−1.28, 2.13
Pop 10 k-100 k	−0.623	0.549	−2.67, 1.42	−0.597	0.567	−2.65, 1.45
Not married	−2.238	**0.001**	−3.54, −0.94	−2.271	**0.001**	−3.57, −0.97
MMI	−0.851	**<0.0001**	−1.00, −0.70	−0.859	**<0.0001**	−1.02, −0.70

D) Analysis of “Life Satisfaction-11” using logistic regression.								
Variables	MMI-30				MMI-25			
	*β*	Value of *p*	OR	95% CI	*β*	Value of *p*	OR	95% CI
Ethnicity, white	0.120	0.333	1.27	0.78, 2.07	0.127	0.308	1.29	0.79, 2.09
Age	0.004	0.440	1.00	0.99, 1.01	0.003	0.602	1.00	0.99, 1.01
Sex, male	−0.140	**0.047**	0.76	0.57, 1.00	−0.139	**0.048**	0.76	0.57, 1.00
Education, high school or greater	0.416	**<0.0001**	2.30	1.58, 3.34	0.419	**<0.0001**	2.31	1.59, 3.36
Married	0.338	**<0.0001**	1.97	1.53, 2.53	0.340	**<0.0001**	1.97	1.53, 2.54
MMI	−0.115	**<0.0001**	0.89	0.87, 0.92	−0.117	**<0.0001**	0.89	0.86, 0.92

E) Analysis of “Quality of Life Score” using logistic regression.								
Variables	MMI-30				MMI-25			
	*β*	Value of *p*	OR	95% CI	*β*	Value of *p*	OR	95% CI
Ethnicity, white	0.347	**0.006**	2.00	1.22, 3.28	0.357	**0.005**	2.04	1.25, 3.34
Age	−0.009	0.081	0.99	0.98, 1.00	−0.011	**0.040**	0.99	0.98, 1.00
Sex, male	−0.173	**0.025**	0.71	0.52, 0.96	−0.173	**0.025**	0.71	0.52, 0.96
Education, high school or greater	0.194	**0.041**	1.47	1.02, 2.14	0.197	**0.039**	1.48	1.02, 2.15
Married	0.355	**<0.0001**	2.04	1.559, 2.67	0.357	**<0.0001**	2.04	1.55, 2.68
MMI	−0.147	**<0.0001**	0.86	0.84, 0.89	−0.153	**<0.0001**	0.86	0.83, 0.89

^*^Baseline is population < 10,000 individuals. Bold font indicates statistical significance.

### Comparison of MMI-30 vs. MMI-25 using the NTSCI dataset

Logistic regression model outcomes were compared between the MMI-30 and MMI-25 using the NTSCI dataset (Table 3). The MMI-30 significantly correlated with HCU-FNCNR, PCS-12, MCS-12, LiSAT-11, and the QoL scores (p < 0.0001 in all models). Similarly, the MMI-25 achieved the same significance in all models (p < 0.0001). The MMI-25 correlation coefficient was larger than the MMI-30 and the odds ratio was slightly stronger than the MMI-30, suggesting that the MMI-25 was as effective as the MMI-30.

**Table 3:** Comparison of MMI-30 vs. MMI-25 and the health outcome measures in participants with non-traumatic spinal cord injury (NTSCI).

A) Analysis of “Healthcare Utilization-Felt that Needed Care was Not Received” using logistic regression.								
Variables	MMI-30				MMI-25			
	*β*	Value of *p*	OR	95% CI	*β*	Value of *p*	OR	95% CI
Live in own home	−0.054	0.686	0.90	0.53, 1.52	−0.057	0.673	0.89	0.53, 1.51
Incomplete SCI	−0.065	0.704	0.88	0.45, 1.71	−0.062	0.716	0.88	0.45, 1.72
Sex, male	−0.431	**0.001**	0.42	0.25, 0.71	−0.419	**0.001**	0.43	0.26, 0.72
Age	−0.006	0.512	0.99	0.98, 1.01	−0.005	0.547	1.00	0.98, 1.01
Days since injury	−0.000	0.483	1.00	1, 1	−0.000	0.492	1.00	1, 1
MMI	0.129	**<0.0001**	1.14	1.08, 1.20	0.135	**<0.0001**	1.14	1.08, 1.21

B) Analysis of “PCS-12” using multiple linear regression.						
Variables	MMI-30			MMI-25		
	*β*	Value of *p*	95% CI	*β*	Value of *p*	95% CI
Age	−0.103	**<0.001**	−0.16, −0.05	−0.108	**<0.0001**	−0.16, −0.05
Sex, male	0.221	0.782	−1.35, 1.79	0.096	0.904	−1.47, 1.66
Incomplete SCI	−0.683	0.526	−2.80, 1.43	−0.724	0.500	−2.83, 1.38
Area of residence*						
Large cities	0.925	0.449	−1.48, 3.35	0.960	0.430	−1.43, 3.35
Pop >100 k	−0.528	0.617	−2.60, 1.55	−0.472	0.654	−2.54, 1.60
Pop 10 k-100 k	−0.024	0.986	−2.73, 2.68	0.043	0.975	−2.66, 2.74
Not married	−0.314	0.710	−1.97, 1.35	−0.333	0.693	−1.99, 1.32
MMI	−0.744	**<0.0001**	−0.91, −0.58	−0.779	**<0.0001**	−0.95, −0.61

C) Analysis of “MCS-12” using multiple linear regression.						
Variables	MMI-30			MMI-25		
	*β*	value of p	95% CI	β	value of p	95% CI
Age	0.024	0.533	−0.05, 0.10	0.018	0.642	−0.06, 0.09
Sex, male	−0.509	0.645	−2.68, 1.66	−0.675	0.542	−2.85, 1.50
Incomplete SCI	−2.347	0.117	−5.28, 0.59	−2.389	0.110	−5.32, 0.54
Area of residence*						
Large cities	1.987	0.241	−1.34, 5.31	2.032	0.230	−1.29, 5.36
Pop >100 k	0.859	0.558	−2.02, 3.74	0.925	0.528	−1.95, 3.80
Pop 10 k-100 k	2.533	0.186	−1.22, 6.29	2.620	0.171	−1.13, 6.37
Not married	−1.467	0.211	−3.77, 0.83	−1.481	0.206	−3.78, 0.82
MMI	−1.036	**<0.0001**	−1.26, −0.81	−1.069	**<0.0001**	−1.30, −0.83

D) Analysis of “Life Satisfaction-11” using logistic regression.								
Variables	MMI-30				MMI-25			
	*β*	Value of *p*	OR	95% CI	*β*	Value of *p*	OR	95% CI
Ethnicity, white	0.081	0.700	1.18	0.52, 2.67	0.088	0.672	1.19	0.53, 2.69
Age	−0.005	0.496	1.00	0.98, 1.01	−0.006	0.421	0.99	0.98, 1.01
Sex, male	−0.130	0.245	0.77	0.50, 1.20	−0.139	0.214	0.76	0.49, 1.17
Education, high school or greater	0.018	0.906	1.04	0.58, 1.87	0.025	0.867	1.05	0.58, 1.89
Married	0.307	**0.009**	1.85	1.16, 2.93	0.306	**0.009**	1.84	1.16, 2.92
MMI	−0.159	**<0.0001**	0.85	0.81, 0.90	−0.159	**<0.0001**	0.85	0.81, 0.90

E) Analysis of “Quality of Life score” using logistic regression.								
Variables	MMI-30				MMI-25			
	*β*	Value of *p*	OR	95% CI	*β*	Value of *p*	OR	95% CI
Ethnicity, white	0.374	0.075	2.11	0.93, 4.80	0.381	0.067	2.14	0.95, 4.83
Age	−0.020	**0.015**	0.98	0.97, 1.00	−0.021	**0.011**	0.98	0.96, 1.00
Sex, male	0.026	0.820	1.05	0.68, 1.64	0.017	0.884	1.03	0.66, 1.61
Education, high school or greater	−0.028	0.856	0.95	0.52, 1.73	−0.022	0.888	0.96	0.52, 1.75
Married	0.300	**0.011**	1.82	1.15, 2.89	0.297	**0.012**	1.81	1.14, 2.87
MMI	−0.169	**<0.0001**	0.85	0.80, 0.89	−0.170	**<0.0001**	0.84	0.80, 0.89

^*^Baseline is population < 10,000 individuals. Bold font indicates statistical significance.

## Discussion

Previously, we reported that multimorbidity using the MMI-30 was associated with higher HCU and lower physical and mental health and QoL in persons with TSCI (11) and NTSCI (13). In this study, we applied three network models: GGM, Ising Model, and MGM to the 2011–2012 Canadian SCI Community Survey dataset (24) and created the MMI-25, a short form of the MMI-30.

Within the TSCI dataset (n = 1,137), the three network models showed medium-dense connections, with most of the associations being positive. Overall results of centrality, correlation stability, and significance testing in all three models indicated stable network structures. Notably, several SHCs were isolated from the networks, which included cancer, diabetes, DVT, heart disease, liver disease, kidney stones, and/or injuries caused by loss of sensation. Strong connections were evident between the QoL score, LiSAT-11, MCS-12, depression, elbow/wrist, and shoulder problems; of which the most significant edge weights were between the QoL score and LiSAT-11, depression and MCS-12, and elbow/wrist and shoulder problems. In alignment with published literature regarding QoL and life satisfaction (5–7) as well as depression and MCS-12 (26, 28, 34) we found strong connections between the QoL score and LiSAT-11 and depression and MCS-12 in the network models. Several factors have high associations with the QoL score and LiSAT-11, including both modifiable and non-modifiable ones, such as pain, contractures, sleep problems, bowel and sexual dysfunction (34). Surprisingly, in our study, these conditions were not found to be strongly connected to the QoL score and LiSAT-11 in the TSCI networks. This could be due to the design of the 2011–2012 Canadian SCI Community Survey (24), the way in which the QoL score was measured (5-point question rating overall QoL and 6-point question asking about overall life satisfaction), or the fact that QoL was self-reported and based on the participants’ account of the past 2 weeks preceding the survey. Future studies should consider using longitudinal domain-specific measures to provide more concrete information on areas of dissatisfaction and guidance for clinical care.

In terms of the strong connection between elbow/wrist problems and shoulder problems and its significant edge weight in both the TSCI and NTSCI networks, this was an unexpected finding when considering all the other possible connections within the 35 nodes. However, as persons with SCI are highly dependent on their arms and hands for mobility and several activities of daily living, they are at high risk for shoulder, elbow, wrist, and hand injuries, including neuromusculoskeletal pathologies and nociceptive pain (35). Shoulder problems can be caused by acute injury or chronic pathology, but are most often related to overuse injuries of the rotator cuff (36–38). Whereas for elbow/wrist problems, the elbow joint is often overused particularly during push-up manoeuvres required for both weight shifts and transfers (39). Both elbow/wrist and shoulder problems can significantly negatively affect a person’s health and function; thus, this significant association between elbow/wrist problems and shoulder problems can enable clinicians to identify these injuries earlier, and employ treatment and/or preventive strategies to preserve shoulder and elbow function after SCI.

Another important observation within the three TSCI network structures and the node centrality measures was the role of AD. The high centrality scores for AD suggested that it plays an important role in connecting several nodes within each network. AD is characterized by the acute elevation of arterial blood pressure and bradycardia in response to stimuli such as urinary retention, constipation, or infection (40, 41). Persons with an SCI above T6 are at high risk of developing AD; moreover, those with complete injuries have a greater likelihood of AD episodes than those with an incomplete injury (42). Left untreated AD may have serious consequences such as stroke, seizures, and cardiac arrest. Our findings here indicate that AD is central to many other SHCs and suggests that if AD can be effectively managed, treated or prevented, then other SHCs such as light headedness, spasticity, and health outcomes such as PCS-12 and MCS-12 may also be improved.

When comparing the network differences between TSCI and NTSCI, the small NTSCI sample size (*n* = 412) resulted in sparse network structures. However, the key associations identified in the TSCI networks were also observed in the NTSCI networks, for example the connections between the QoL score and LiSAT-11 and elbow/wrist problems and shoulder problems. Thus, rather than creating two network-derived MMIs, one for TSCI and one for NTSCI, we chose to create one generalized MMI for both types of SCI. To do this, we reviewed the bivariate and network results, and removed five SHCs (cancer, diabetes, DVT, high blood pressure and liver disease) from the MMI-30, creating the network-derived MMI-25.

Logistic regression models were constructed to examine the MMI-25’s influence on each health outcome measure, then both the MMI-25 and the original MMI-30 were compared. Our findings indicated that the MMI-25 was as effective as the MMI-30, as it demonstrated the same significance and a larger correlation coefficient. Accordingly, the MMI-25 would be easier for clinicians to incorporate into their routine work to determine patients’ risk for poorer health outcomes (as evident in the regression models no information was lost).

Several limitations of this study should be considered. First, the data in the 2011–2012 Canadian SCI Community Survey is self-reported, which may be subject to recall bias. Second, cross-sectional data cannot be used to infer causality, it is not clear to determine if the most central symptom caused other symptoms/outcomes, the other way around, or both. Thus, future research should consider conducting a longitudinal SCI survey. Third, the NTSCI sample size of 412 participants, while relatively large compared to other NTSCI studies, resulted in sparse and unstable network structures, limiting the ability to detect differences between centrality estimates and estimation accuracy. Fourth, for the Ising Model, the two continuous independent variables (PCS-12 and MCS-12 scores) were not included; therefore, some of the information related to the connections between the 30 SHCs and these two continuous outcomes may not be measured. Fifth, the GGM requires variables to have multivariate Gaussian distribution, which is not the case for most variables in our study, and the efficiency of GGM may be affected by including binary variables. However, this limitation was not a problem for MGM which used both continuous and discrete variables. Nevertheless, these study results illustrate the value of network analysis in SCI outcome research.

To our knowledge, this is the first study to perform network analysis on SHCs and health outcomes in persons with SCI. Network analysis provided another way to examine the relationship between multimorbidity and health outcomes compared with the traditional statistical methods. The results demonstrated strong connections between (1) the QoL score and LiSAT-11, (2) MCS-12 and depression, and (3) elbow/wrist problems and shoulder problems, within the network structures. Furthermore, cancer, DVT, diabetes, high blood pressure and liver disease were isolated. Thus, the network-derived MMI consisted of 25 SHCs, and was shown to be as powerful as the previously published MMI-30 (11, 13). This study used cross-sectional data, but network analysis can also be applied to longitudinal data and may be a topic for future analysis. Future directions include piloting the MMI-25 as a screening tool to identify patients at risk of having poor health outcomes during routine community follow-up and conducting additional psychometric analyses using longitudinal data.

## Data availability statement

The datasets presented in this article are not readily available because participants did not provide consent to share the data. Requests to access the datasets should be directed to the corresponding author.

## Ethics statement

The studies involving humans were approved by an independent Canadian Independent Review Board (IRB), the Research Ethics Board of Université Laval and local IRBs. The studies were conducted in accordance with the local legislation and institutional requirements. The participants provided their written informed consent to participate in this study. Written informed consent was obtained from the individual (s) for the publication of any potentially identifiable images or data included in this article.

## Author contributions

NF: Conceptualization, Formal analysis, Writing – original draft. HH: Formal analysis, Conceptualization, Writing – review & editing. DW: Formal analysis, Writing – original draft. SH: Writing – review & editing. JP: Writing – review & editing. KW: Writing – review & editing. JS: Writing – review & editing. RC-M: Writing – review & editing. CLC: Writing – review & editing. CJC: Writing – review & editing. VN: Funding acquisition, Writing – review & editing.
