# Supplementary material for: Network analysis of multimorbidity and health outcomes among persons with spinal cord injury in Canada
Source: Front Neurol. 2024 Jan 5;14:1286143. doi: 10.3389/fneur.2023.1286143 (PMC10797060; doi:10.3389/fneur.2023.1286143)
Supplement: Supplementary file 1 [file Data_Sheet_1.docx]

Supplementary Material

Supplementary Table 1

Bivariate analysis of the 30 secondary health conditions (SHCs) and the health outcome measures [Healthcare Utilization-Felt Needed Care was Not Received (HCU-FNCNR), Physical Component Summary (PCS-12) score, Mental Component Summary (MCS-12) score, Life Satisfaction-11 (LiSAT-11) score, and Quality of Life (QoL) score] in persons with traumatic spinal cord injury (TSCI) and non-traumatic spinal cord injury (NTSCI).

| **SHCs** | **TSCI Health Outcomes** | | | | | **NTSCI Health Outcomes** | | | | |
| --- | --- | --- | --- | --- | --- | --- | --- | --- | --- | --- |
|  | **HCU-FNCNR** | **PCS-12** | **MCS-12** | **LiSAT-11** | **QoL** | **HCU-**  **FNCNR** | **PCS-**  **12** | **MCS-**  **12** | **LiSAT-**  **11** | **QoL** |
| Autonomic dysreflexia | <.0001 | <.0001 | <.0001 | 0.000 | <.0001 | 0.100 | 0.002 | <.0001 | 0.001 | 0.001 |
| Bowel incontinence | 0.001 | 0.001 | 0.995 | 0.393 | 0.580 | 0.784 | 0.031 | 0.033 | 0.004 | 0.031 |
| Cancer | 0.233 | 0.002 | 0.159 | 0.066 | 0.009 | 0.788 | 0.183 | 0.111 | 0.992 | 0.149 |
| Constipation | <.0001 | <.0001 | <.0001 | <.0001 | 0.000 | 0.038 | 0.001 | 0.064 | 0.020 | 0.028 |
| Deep vein thrombosis/pulmonary embolism | 0.642 | 0.186 | 0.139 | 0.178 | 0.814 | 1 | 0.941 | 0.136 | 0.031 | 0.151 |
| Depression/mood problem | <.0001 | <.0001 | <.0001 | <.0001 | <.0001 | 0.171 | 0.093 | <.0001 | 0.000 | <.0001 |
| Diabetes | 0.272 | 0.231 | 0.431 | 0.776 | 0.290 | 0.531 | 0.088 | 0.358 | 0.057 | 0.341 |
| Elbow/wrist problems | 0.000 | <.0001 | 0.002 | 0.628 | 0.022 | 0.072 | <.0001 | 0.001 | 0.033 | 0.021 |
| Fatigue | <.0001 | <.0001 | <.0001 | <.0001 | <.0001 | 0.010 | <.0001 | <.0001 | <.0001 | <.0001 |
| Heart disease | 0.007 | 0.169 | 0.003 | 0.049 | 0.014 | 0.500 | 0.062 | 0.750 | 0.810 | 0.007 |
| High blood pressure | 0.010 | 0.002 | <.0001 | 0.026 | 0.003 | 0.428 | 0.211 | 0.487 | 0.521 | 0.208 |
| Injuries caused by loss of sensation | <.0001 | <.0001 | <.0001 | <.0001 | <.0001 | 0.000 | 0.001 | 0.000 | 0.003 | 0.096 |
| Joint contractures | 0.002 | <.0001 | <.0001 | <.0001 | <.0001 | 0.000 | <.0001 | 0.001 | 0.001 | 0.000 |
| Kidney stones | 0.007 | 0.000 | 0.526 | 0.053 | 0.009 | 0.145 | 0.314 | 0.001 | 0.111 | 0.092 |
| Light headedness/dizziness | <.0001 | <.0001 | <.0001 | <.0001 | <.0001 | 0.001 | <.0001 | <.0001 | 0.000 | 0.001 |
| Liver disease | 0.031 | 0.111 | <.0001 | 0.019 | 0.009 | 1 | 0.871 | 0.107 | 0.738 | 1 |
| Neurological deterioration | <.0001 | <.0001 | <.0001 | 0.001 | <.0001 | 0.013 | <.0001 | <.0001 | <.0001 | 0.000 |
| Neuropathic pain | 0.000 | <.0001 | <.0001 | <.0001 | <.0001 | <.0001 | <.0001 | 0.000 | 0.000 | 0.004 |
| Osteoarthritis/degenerative arthritis | 0.002 | <.0001 | 0.032 | 0.110 | 0.030 | 0.003 | 0.004 | 0.226 | 0.657 | 0.271 |
| Osteoporosis | 0.089 | 0.000 | 0.920 | 0.037 | 0.977 | 0.007 | 0.489 | 0.030 | 0.510 | 0.126 |
| Pressure ulcers | 0.004 | <.0001 | 0.003 | 0.005 | 0.003 | 0.049 | 0.239 | 0.007 | 0.033 | 0.021 |
| Respiratory infections | 0.000 | <.0001 | 0.002 | 0.042 | 0.184 | 0.083 | 0.006 | <.0001 | 0.007 | <.0001 |
| Sexual dysfunction | 0.025 | <.0001 | 0.001 | 0.005 | 0.001 | 0.997 | 0.007 | 0.000 | <.0001 | 0.029 |
| Shoulder problems | 0.004 | <.0001 | 0.015 | 0.310 | 0.137 | 0.065 | <.0001 | 0.169 | 0.001 | 0.002 |
| Spasticity | 0.100 | <.0001 | 0.702 | 0.551 | 0.535 | 0.405 | 0.000 | 0.004 | 0.005 | 0.020 |
| Trouble sleeping | 0.000 | <.0001 | <.0001 | 0.000 | <.0001 | 0.002 | <.0001 | <.0001 | <.0001 | <.0001 |
| Ulcer/gastric esophageal reflux disease | 0.000 | <.0001 | 0.001 | 0.135 | 0.028 | 0.001 | 0.020 | 0.043 | 0.176 | 0.022 |
| Urinary incontinence | <.0001 | <.0001 | 0.004 | 0.234 | 0.385 | 0.208 | 0.000 | 0.050 | 0.084 | 0.125 |
| Urinary tract infection | 0.001 | <.0001 | 0.209 | 0.217 | 0.813 | 0.470 | 0.032 | 0.005 | 0.027 | 0.158 |
| Weight problem | <.0001 | <.0001 | <.0001 | <.0001 | 0.001 | 0.010 | <.0001 | 0.002 | 0.005 | 0.001 |

Note: Grey highlights indicate non-significance across the health outcome measures.

# Supplementary Information on Network Models

**Comparison of Gaussian Graphical Models, Ising Models, and Mixed Graphical Models on their Information and Reliability.**

**1. Gaussian Graphical Model (GGM)**:

***Information Obtained***: GGMs are commonly used for modeling relationships between continuous variables. They provide insight into conditional dependencies and independencies between variables in the form of a precision (inverse covariance) matrix. This can reveal which variables are directly or indirectly connected in a network.

***Reliability***: GGMs assume multivariate Gaussian distributions, which may not hold for all types of data. They work best when variables are approximately normally distributed and linearly related. Inaccurate distributional assumptions can lead to unreliable results.

**2. Ising Model:**

***Information Obtained***: Ising Models are used for binary or categorical data, making them suitable for modeling binary interactions or states (e.g., presence/absence, like/dislike). They provide information about pairwise associations and the strength and sign of interactions.

***Reliability***: Ising Models are robust for binary data, but they include simplifications, such as assuming pairwise interactions only. They may not capture higher-order interactions or nonlinear relationships, potentially limiting their applicability.

**3. Mixed Graphical Model (MGM):**

***Information Obtained***: MGMs combine continuous and categorical variables in a single network, offering insights into both types of relationships. They can capture both conditional dependencies among continuous variables (like GGMs) and pairwise associations among categorical variables (like Ising Models).

***Reliability***: The reliability of MGMs depends on the quality and appropriateness of the data and the underlying assumptions. As with any mixed-model approach, care must be taken to ensure that the combination of continuous and categorical data is justified and the model assumptions are met.

In summary, the choice among the GGM, Ising, and MGM depends on the nature of data and the type of research questions. Each model has its strengths and limitations, and their reliability hinges on the appropriateness of their assumptions and the quality of the data being analyzed. It is often advisable to consider the specific characteristics of dataset and the relationships between variables when selecting the most suitable network model. For our specific application, the MGM was the best model for our data.
